# Association Between Second-hand Smoke Exposure and Depressive Symptoms Among Japanese Adults: A Cross-sectional Study

**DOI:** 10.2188/jea.JE20190146

**Published:** 2020-12-05

**Authors:** Taiji Noguchi, Hiroko Nakagawa-Senda, Yuya Tamai, Takeshi Nishiyama, Miki Watanabe, Akihiro Hosono, Kiyoshi Shibata, Mari Ichikawa, Ryozo Wakabayashi, Kenji Nagaya, Kanae Ema, Naoko Okamoto, Shoko Tsujimura, Hitomi Fujita, Mayumi Kamiya, Fumi Kondo, Tamaki Yamada, Sadao Suzuki

**Affiliations:** 1 Department of Public Health, Nagoya City University Graduate School of Medical Sciences, Nagoya, Japan; 2 Department of Social Science, Center for Gerontology and Social Science, National Center for Geriatrics and Gerontology, Aichi, Japan; 3 Ministry of Health, Labour and Welfare, Tokyo, Japan; 4 Atsuta Public Health Center, City of Nagoya, Nagoya, Japan; 5 Department of Health and Nutritional Sciences, Nagoya Keizai University, Aichi, Japan; 6 Department of Health Nutritional Sciences, Osaka Shoin Women’s University, Osaka, Japan; 7 Department of Health Sciences, Toyohashi Sozo University, Aichi, Japan; 8 Department of Health Sciences, Nihon Fukushi University, Aichi, Japan; 9 Department of Nursing, Chukyo Gakuin University, Gifu, Japan; 10 Okazaki City Medical Association, Public Health Center, Aichi, Japan

**Keywords:** depressive symptoms, Japanese adults, second-hand smoke exposure

## Abstract

**Background:**

Second-hand smoke exposure has been associated with poor mental health. However, among Japanese adults, little is known about the association between second-hand smoking and depressive symptoms. We examined this association in a cross-sectional study among a Japanese general adult population sample.

**Methods:**

Japanese adults were recruited from the Japan Multi-Institutional Collaborative Cohort Study in the Okazaki area between 2012 and 2017. Second-hand smoke exposure and smoking status were assessed using a self-administered questionnaire. Based on their frequency of exposure to second-hand smoke, non-smokers and smokers were categorized as “almost never,” “sometimes,” and “almost every day”. Depressive symptoms were defined by a Kessler 6 score ≥5 points. We performed a multivariable Poisson regression analysis to obtain adjusted prevalence ratios (PRs) and 95% confidence intervals (CIs) for depressive symptoms.

**Results:**

Overall, 5,121 participants (4,547 non-smokers and 574 smokers) were included whose mean age was 63.6 (standard deviation [SD], 10.3) years for non-smokers and 59.33 (SD, 10.2) years for smokers. The association between second-hand smoking and depressive symptoms was significant among non-smokers, but not among smokers. Among non-smokers, PRs compared with “almost never” were 1.25 (95% CI, 1.09–1.42) for “sometimes” and 1.41 (95% CI, 1.09–1.84) for “almost every day” (*P* for trend <0.001); among smokers, PRs compared with “almost never” were 1.30 (95% CI, 0.82–2.06) for “sometimes” and 1.44 (95% CI, 0.90–2.33) for “almost every day” (*P* for trend = 0.144).

**Conclusions:**

Second-hand smoking and depressive symptoms were associated among non-smokers. Our findings indicate the importance of tobacco smoke control for mental health.

## INTRODUCTION

Second-hand smoke exposure poses a serious burden on population health. An estimated 603,000 deaths, approximately 1.0% of worldwide mortality, were attributed to second-hand smoking^1^; disability-adjusted life-years (DALYs) lost due to second-hand smoking were 10.9 million, which accounted for about 0.7% of the total DALYs lost due to disease.^1^ There is robust evidence indicating a causal relationship between second-hand smoking and various diseases, such as coronary artery disease, lung cancer, and stroke.^2^^–^^4^ As there is no safe level of exposure to second-hand smoke according to the mechanisms of second-hand smoking and respiratory diseases,^5^ the reduction of second-hand smoking is an urgent public health need.

Meanwhile, several epidemiological studies have pointed to the possible negative impact of second-hand smoking on mental health among pregnant women,^6^ children,^7^^,^^8^ and adults.^9^^–^^13^ Zeng et al, who conducted a meta-analysis that integrated 11 cross-sectional studies, revealed that second-hand smoking was positively and significantly associated with depressive symptoms and psychological distress.^14^ Correspondingly, exposure to second-hand smoke has been found to increase the risk of depressive symptoms and strengthen the association between smoking and poor mental health.^15^ Second-hand smoking has been considered as a potential factor that harms mental health through decreased sensitivity to pleasure due to nicotine intake^16^ and increased psychological stress.^17^ However, previous cross-sectional^18^ and longitudinal^19^ studies that measured biomarkers of second-hand smoke exposure did not find an association; therefore, there is no current consensus in the literature.

A “Report of the Study Group on the Health Effects of Smoking” by the Japanese Ministry of Health, Labour and Welfare has shown that the effects of second-hand smoking on poor mental health outcomes, such as depression, are possible, but there is not enough supporting evidence.^4^ Among Japanese adults, there has been only one report on the association between second-hand smoking and mental health among working people,^13^ and there have been no studies of the general population. Evidence for Japanese adults is lacking. Therefore, we aimed to examine the relationship between second-hand smoke exposure and depressive symptoms among a Japanese general adult population sample.

## MATERIAL AND METHODS

### Study population

The Japan Multi-Institutional Collaborative Cohort Study (J-MICC Study) was initiated in 2005 with the aim of obtaining fundamental data for the prevention of lifestyle-related diseases, mainly cancer, taking genetic traits into account.^20^^,^^21^ The present cross-sectional study enrolled Japanese adults who participated in the J-MICC Study in the Okazaki area. Specifically, we collected the data of 5,321 individuals (out of the total 7,580 who had been asked to participate; response rate: 70.2%) who responded to the J-MICC questionnaire (either in person or by mail) when visiting the Okazaki Medical Association Public Health Center in Aichi Prefecture, Japan, between 2013 and 2017. We excluded respondents who did not respond to the items pertaining to depressive symptoms (95 respondents) and smoking history and second-hand smoking (105 respondents). Finally, we analyzed the data of 5,121 respondents (valid response rate: 96.2%). All participants in the present study provided written informed consent, and the study protocol was approved by the ethics committee of the Nagoya City University Graduate School of Medicine. The study was conducted in accordance with the guidelines of the Declaration of Helsinki.

### Depressive symptoms

We assessed depressive symptoms using the Kessler 6 scale (K6),^22^ Japanese version,^23^ which consists of six items asking how frequently respondents have experienced symptoms of psychological distress during the past 30 days.^24^ The response options range from 0 = none of the time to 4 = all of the time (possible total score range: 0–24). Based on a previous study,^25^ we used the K6 scale to measure depressive symptoms. A Canadian study showed that the K6 scale is a valid tool for assessing depressive symptoms (areas under the curve and 95% confidence interval [CI] were 0.93 and 0.91–0.95, respectively).^25^ Following this previous study,^25^ we classified respondents with K6 scores ≥5 as having depressive symptoms. This cut-off point has been shown a sensitivity of 0.92 (95% CI, 0.88–0.95) and a specificity of 0.78 (95% CI, 0.77–0.79) for depressive symptoms.

### Second-hand smoke exposure

Exposure frequency to second-hand smoking was assessed using a single question: “Have you inhaled others’ (ie, smokers) cigarette smoke at home, in an office, or elsewhere in the last year?”, with the following six response options: “almost never”, “sometimes”, “almost every day, 2 hours/day or less”, “almost every day, 2 to 4 hours/day”, “almost every day, 4 to 6 hours/day”, and “almost every day, 6 hours/day or longer”. In the present analysis, respondents who answered “almost every day, 2 hours/day or less”, “almost every day, 2 to 4 hours/day”, “almost every day, 4 to 6 hours/day”, or “almost every day, 6 hours/day or longer” were combined into the group “almost every day” because of the small number of respondents. Additionally, smoking status was assessed using the following question: “Are you a current, former, or never smoker?”. We combined the respondents who answered “former” and “never” and categorized smoking status into two groups, namely “non-smoker” and “smoker”. Accordingly, we classified the frequency of second-hand smoke exposure into three groups, namely “almost never”, “sometimes”, and “almost every day” according to non-smoker and smoker status, respectively.

### Covariates

Questions on socio-demographic characteristics, health status, and lifestyle were included in the analyses as covariates. These covariates included age, gender, body mass index (BMI), educational attainment, marital status, employment status, present illness (cancer, heart disease, and stroke), alcohol consumption, smoking volume, and total physical activity. Age was grouped into the following seven categories: under 44, 45–49, 50–54, 55–59, 60–64, 65–69, and 70 years or older. BMI was categorized as follows: <18.5, 18.5–24.9, and ≥25 kg/m^2^. Educational attainment was categorized as follows: <10, 10–12, and ≥13 years. Marital status was categorized as follows: married, divorced, separated, and never married. Employment status was categorized as follows: regular employment, non-regular employment, other, and not employed. Present illness was assessed using a questionnaire that asked respondents whether they had received cancer, heart disease, and/or stroke diagnoses. Respondents were required to select “yes” or “no” responses. Alcohol consumption was assessed for current drinkers (defined as those who had consumed alcohol at least once a week during the last year) based on the reported consumption frequency and was categorized as follows: “never”, “1–3 days/month”, “1–6 days/week”, and “every day”. Smoking volume was assessed using the number of cigarettes smoked per day and the smoking period. Then, pack-years (the number of cigarettes smoked per day ÷ 20 cigarettes × the smoking period) were calculated. Smoking volume was categorized as follows: “0”, “1–19 pack(s) per year”, “20–39 packs per year”, and “over 40 packs per year”. Total physical activity was estimated as metabolic equivalent hours per day according to the frequency and duration of daily and leisure time activities.^26^ We divided participants into tertiles based on their total physical activity levels.

### Statistical analysis

All analyses were stratified by smoking status (non-smokers and smokers). First, we calculated descriptive statistics and investigated differences in each variable based on the frequency of second-hand smoke exposure. Second, to examine the association between second-hand smoking and depressive symptoms, we conducted a multivariable Poisson regression analysis with robust standard errors and obtained prevalence ratios (PRs) and 95% CIs for depressive symptoms. As the percentage of individuals with depressive symptoms (about 26.0%) was >10%, adjusted odds ratios derived from logistic regression could no longer approximate the PR.^27^ The analyses were adjusted for age and gender (model 1). Next, we added BMI, educational attainment, marital status, employment status, present illness (cancer, heart disease, and stroke), alcohol consumption, smoking volume, and total physical activity to model 1 (model 2). Third, to eliminate the influence of the working conditions, we performed stratified analysis according to employment status (regular employment, non-regular employment, other, and not employed), adjusted by all covariates. To avoid a small sample size for each group, the analyses were performed by combining non-smokers and smokers.

To mitigate potential biases caused by missing information, we used the multiple imputation approach, under the missing at random (MAR) assumption (ie, the missing data mechanism depends only on the observed variables). We generated 20 imputed datasets using the Multiple Imputation by Chained Equations (MICE) procedure and pooled the results using the standard Rubin’s rule.^28^

The significance level was set at <0.05. We used R (Version 3.4.3 for Windows; R Foundation for Statistical Computing, Vienna, Austria) for all statistical analyses. The multiple imputation approach involved the use of the mice function (mice package).

## RESULTS

A total of 5,121 respondents (2,891 males and 2,230 females) were included in the final analysis. Table 1 shows the respondents’ characteristics. The number of non-smokers and smokers were 4,547 and 574, respectively. The mean age was 63.6 (SD, 10.3) years for non-smokers and 59.33 (SD, 10.2) years for smokers. Regarding the frequency of second-hand smoke exposure, 3,168 (69.8%) non-smokers responded “almost never”, 1,202 (26.4%) responded “sometimes”, and 177 (3.9%) responded “almost every day”. Among smokers, 158 (27.5%) responded “almost never”, 243 (42.3%) responded “sometimes”, and 173 (30.1%) responded “almost every day”. Among non-smokers, respondents with the highest frequency of second-hand smoking tended to be under 65 years old, women, married, regular employees, have a high BMI, not have cancer, have low levels of alcohol consumption, and have depressive symptoms. Smokers tended to be similar to non-smokers, except for present illness, alcohol consumption, and total physical activity. Smokers with high frequencies of second-hand smoking tended not to have cancer, have heart disease, and have lower total physical activity levels.

**Table 1.** Respondent characteristics

			Non-smokers (*n* = 4,547)			Smokers (*n* = 574)		
	
			Second-hand smoking			Second-hand smoking		
	
			Almost never, *n* (%)	Sometimes, *n* (%)	Almost every day, *n* (%)	Almost never, *n* (%)	Sometimes, *n* (%)	Almost every day, *n* (%)
	
			*n* = 3,168	*n* = 1,202	*n* = 177	*n* = 158	*n* = 243	*n* = 173
Age, years		Under 44	128 (4.0)	75 (6.2)	21 (11.9)	10 (6.3)	23 (9.5)	23 (13.3)
		45 to 49	193 (6.1)	116 (9.7)	18 (10.2)	9 (5.7)	22 (9.1)	27 (15.6)
		50 to 54	268 (8.5)	159 (13.2)	29 (16.4)	25 (15.8)	23 (9.5)	29 (16.8)
		55 to 59	284 (9.0)	146 (12.1)	26 (14.7)	22 (13.9)	37 (15.2)	42 (24.3)
		60 to 64	374 (11.8)	160 (13.3)	35 (19.8)	18 (11.4)	40 (16.5)	18 (10.4)
		65 to 69	695 (21.9)	242 (20.1)	27 (15.3)	33 (20.9)	44 (18.1)	20 (11.6)
		70 or older	1,226 (38.7)	304 (25.3)	21 (11.9)	41 (25.9)	54 (22.2)	14 (8.1)
Gender		Males	1,645 (51.9)	702 (58.4)	60 (33.9)	137 (86.7)	215 (88.5)	132 (76.3)
		Females	1,523 (48.1)	500 (41.6)	117 (66.1)	21 (13.3)	28 (11.5)	41 (23.7)
Body mass index, kg/m^2^		<18.5	161 (5.1)	57 (4.7)	12 (6.8)	10 (6.3)	8 (3.3)	11 (6.4)
		18.5 to 24.9	2,367 (74.7)	843 (70.1)	126 (71.2)	113 (71.5)	176 (72.4)	118 (68.2)
		≥25.0	615 (19.4)	289 (24.0)	38 (21.5)	34 (21.5)	57 (23.5)	41 (23.7)
		Missing	25 (0.8)	13 (1.1)	1 (0.6)	1 (0.6)	2 (0.8)	3 (1.7)
Educational attainment, years		<10	430 (13.6)	161 (13.4)	20 (11.3)	19 (12.0)	22 (9.1)	18 (10.4)
		10 to 12	1,306 (41.2)	496 (41.3)	82 (46.3)	67 (42.4)	113 (46.5)	78 (45.1)
		≥13	1,429 (45.1)	545 (45.3)	75 (42.4)	72 (45.6)	108 (44.4)	77 (44.5)
		Missing	3 (0.1)	0 (0.0)	0 (0.0)	0 (0.0)	0 (0.0)	0 (0.0)
Marital status		Married	2,653 (83.7)	990 (82.4)	157 (88.7)	130 (82.3)	211 (86.8)	146 (84.4)
		Divorced	269 (8.5)	79 (6.6)	6 (3.4)	8 (5.1)	11 (4.5)	7 (4.0)
		Separated	107 (3.4)	57 (4.7)	7 (4.0)	12 (7.6)	9 (3.7)	7 (4.0)
		Never married	103 (3.3)	68 (5.7)	4 (2.3)	6 (3.8)	11 (4.5)	11 (6.4)
		Missing	36 (1.1)	8 (0.7)	3 (1.7)	2 (1.3)	1 (0.4)	2 (1.2)
Employment status		Regular employment	920 (29.0)	513 (42.7)	102 (57.6)	72 (45.6)	150 (61.7)	121 (69.9)
		Non-regular employment	427 (13.5)	239 (19.9)	35 (19.8)	14 (8.9)	24 (9.9)	23 (13.3)
		Other	183 (5.8)	52 (4.3)	7 (4.0)	11 (7.0)	16 (6.6)	5 (2.9)
		Non	1,628 (51.4)	395 (32.9)	32 (18.1)	61 (38.6)	53 (21.8)	23 (13.3)
		Missing	10 (0.3)	3 (0.2)	1 (0.6)	0 (0.0)	0 (0.0)	1 (0.6)
Present illness	Cancer	No	2,586 (81.6)	1,028 (85.5)	159 (89.8)	140 (88.6)	208 (85.6)	152 (87.9)
		Yes	359 (11.3)	118 (9.8)	10 (5.6)	11 (7.0)	24 (9.9)	10 (5.8)
		Missing	223 (7.0)	56 (4.7)	8 (4.5)	7 (4.4)	11 (4.5)	11 (6.4)
	Heart disease	No	2,898 (91.5)	1,106 (92.0)	165 (93.2)	151 (95.6)	229 (94.2)	162 (93.6)
		Yes	156 (4.9)	65 (5.4)	10 (5.6)	3 (1.9)	11 (4.5)	6 (3.5)
		Missing	114 (3.6)	31 (2.6)	2 (1.1)	4 (2.5)	3 (1.2)	5 (2.9)
	Stroke	No	2,881 (90.9)	1,111 (92.4)	168 (94.9)	149 (94.3)	229 (94.2)	163 (94.2)
		Yes	118 (3.7)	42 (3.5)	4 (2.3)	4 (2.5)	3 (1.2)	4 (2.3)
		Missing	169 (5.3)	49 (4.1)	5 (2.8)	5 (3.2)	11 (4.5)	6 (3.5)
Alcohol consumption		None	1,564 (49.4)	532 (44.3)	96 (54.2)	44 (27.8)	83 (34.2)	48 (27.7)
		One to three days/month	284 (9.0)	139 (11.6)	11 (6.2)	27 (17.1)	22 (9.1)	16 (9.2)
		One to six days/week	759 (24.0)	317 (26.4)	44 (24.9)	25 (15.8)	66 (27.2)	39 (22.5)
		Everyday	547 (17.3)	211 (17.6)	26 (14.7)	62 (39.2)	72 (29.6)	70 (40.5)
		Missing	14 (0.4)	3 (0.2)	0 (0.0)	0 (0.0)	0 (0.0)	0 (0.0)
Smoking history		Never	2,092 (66.0)	694 (57.7)	120 (67.8)	0 (0.0)	0 (0.0)	0 (0.0)
		Past	1,076 (34.0)	508 (42.3)	57 (32.2)	0 (0.0)	0 (0.0)	0 (0.0)
		Current	0 (0.0)	0 (0.0)	0 (0.0)	158 (100.0)	243 (100.0)	173 (100.0)
Smoking volume, pack-years		0	2,109 (66.6)	700 (58.2)	120 (67.8)	0 (0.0)	0 (0.0)	0 (0.0)
		1 to 19	403 (12.7)	183 (15.2)	19 (10.7)	37 (23.4)	69 (28.4)	42 (24.3)
		20 to 39	314 (9.9)	162 (13.5)	22 (12.4)	78 (49.4)	85 (35.0)	79 (45.7)
		40 over	261 (8.2)	118 (9.8)	11 (6.2)	42 (26.6)	85 (35.0)	49 (28.3)
		Missing	81 (2.6)	39 (3.2)	5 (2.8)	1 (0.6)	4 (1.6)	3 (1.7)
Total physical activity		First tertile (lowest)	866 (27.3)	387 (32.2)	49 (27.7)	58 (36.7)	100 (41.2)	74 (42.8)
		Second tertile	984 (31.1)	356 (29.6)	50 (28.2)	35 (22.2)	69 (28.4)	39 (22.5)
		Third tertile (highest)	967 (30.5)	346 (28.8)	57 (32.2)	51 (32.3)	60 (24.7)	46 (26.6)
		Missing	351 (11.1)	113 (9.4)	21 (11.9)	14 (8.9)	14 (5.8)	14 (8.1)
Depressive symptoms		No	2,428 (76.6)	829 (69.0)	112 (63.3)	123 (77.8)	182 (74.9)	113 (65.3)
		Yes	740 (23.4)	373 (31.0)	65 (36.7)	35 (22.2)	61 (25.1)	60 (34.7)

Table 2 shows the association between second-hand smoking and depressive symptoms among non-smokers and smokers, respectively. Model 1 showed the results of Poisson regression analysis adjusted for age and gender. Among non-smokers, PRs compared with “almost never” were 1.26 (95% CI, 1.11–1.43) for “sometimes” and 1.33 (95% CI, 1.03–1.72) for “almost every day”; among smokers, PRs compared with “almost never” were 1.23 (95% CI, 0.80–1.88) for “sometimes” and 1.43 (95% CI, 0.92–2.22) for “almost every day”. Exposure to second-hand smoke was significantly associated with depressive symptoms among non-smokers (*P* for trend <0.001) but not significantly among smokers (*P* for trend = 0.113). Model 2 showed the results adjusted for the full set of covariates, which showed a similar trend to model 1. Among non-smokers, PRs were 1.25 (95% CI, 1.09–1.42) for “sometimes” and 1.41 (95% CI, 1.09–1.84) for “almost every day”; among smokers, PRs were 1.30 (95% CI, 0.82–2.06) for “sometimes” and 1.44 (95% CI, 0.90–2.33) for “almost every day”. Similar to model 1, exposure to second-hand smoke was significantly associated with depressive symptoms among non-smokers (*P* for trend <0.001) but not significantly among smokers (*P* for trend = 0.144).

**Table 2.** PRs and 95% CIs for depressive symptoms determined by multivariable Poisson regression analysis among non-smokers and smokers

			Non-smoker (*n* = 4,547)				Smoker (*n* = 574)			
	
			Model 1		Model 2		Model 1		Model 2	
			
			PR (95% CI) adjusted by age and gender		PR (95% CI) adjusted by all variables		PR (95% CI) adjusted by age and gender		PR (95% CI) adjusted by all variables	
Second-hand smoking		Almost never	Reference		Reference		Reference		Reference	
		Sometimes	1.26	(1.11–1.43)^**^	1.25	(1.09–1.42)^**^	1.23	(0.80–1.88)	1.30	(0.82–2.06)
		Almost every day	1.33	(1.03–1.72)^*^	1.41	(1.09–1.84)^**^	1.43	(0.92–2.22)	1.44	(0.90–2.33)
			*P* for trend <0.001		*P* for trend <0.001		*P* for trend = 0.113		*P* for trend = 0.144	
Age, years		Under 44	Reference		Reference		Reference		Reference	
		45 to 49	1.10	(0.83–1.46)	1.13	(0.85–1.51)	1.08	(0.57–2.03)	1.08	(0.56–2.08)
		50 to 54	1.07	(0.82–1.40)	1.11	(0.85–1.46)	1.12	(0.62–2.03)	1.17	(0.62–2.20)
		55 to 59	0.90	(0.68–1.18)	0.97	(0.73–1.29)	1.07	(0.60–1.89)	1.05	(0.57–1.94)
		60 to 64	0.83	(0.64–1.09)	0.85	(0.64–1.13)	0.52	(0.25–1.09)^†^	0.56	(0.25–1.28)
		65 to 69	0.58	(0.45–0.76)^**^	0.61	(0.45–0.82)^**^	0.86	(0.46–1.60)	0.84	(0.38–1.88)
		70 or older	0.62	(0.48–0.80)^**^	0.64	(0.47–0.86)^**^	0.66	(0.35–1.27)	0.64	(0.27–1.49)
Gender		Male	Reference		Reference		Reference		Reference	
		Female	1.19	(1.05–1.34)^**^	1.12	(0.95–1.32)	1.39	(0.95–2.04)^†^	1.29	(0.80–2.09)
Body mass index, kg/m^2^		18.5 to 24.9			Reference				Reference	
		<18.5			1.16	(1.00–1.34)^*^			0.83	(0.54–1.28)
		≥25.0			1.27	(1.00–1.62)^†^			1.68	(0.92–3.04)^†^
Educational attainment, years		<10			Reference				Reference	
		10 to 12			0.96	(0.77–1.18)			0.71	(0.41–1.22)
		≥13			0.98	(0.79–1.21)			0.61	(0.34–1.07)^†^
Marital status		Married			Reference				Reference	
		Divorced			1.12	(0.84–1.48)			0.89	(0.41–1.91)
		Separated			1.18	(0.93–1.49)			0.76	(0.30–1.96)
		Never married			1.14	(0.87–1.50)			1.07	(0.53–2.17)
Employment status		Regular employment			Reference				Reference	
		Not-regular employment			1.07	(0.90–1.28)			0.74	(0.37–1.46)
		Other			1.04	(0.78–1.40)			1.20	(0.56–2.54)
		Not employed			1.05	(0.88–1.27)			0.93	(0.51–1.71)
Present illness	Cancer	No			Reference				Reference	
		Yes			1.08	(0.88–1.33)			1.13	(0.59–2.14)
	Heart disease	No			Reference				Reference	
		Yes			1.01	(0.75–1.36)			1.27	(0.55–2.95)
	Stroke	No			Reference				Reference	
		Yes			1.11	(0.80–1.53)			0.98	(0.30–3.15)
Alcohol consumption		Non			Reference				Reference	
		One to three days/month			1.02	(0.84–1.24)			1.02	(0.56–1.85)
		One to six days/week			0.95	(0.82–1.11)			1.18	(0.75–1.85)
		Everyday			0.90	(0.75–1.10)			0.93	(0.60–1.44)
Smoking volume, pack-years		0			Reference					
		1 to 19			1.14	(0.94–1.37)			Reference	
		20 to 39			0.85	(0.67–1.08)			0.89	(0.59–1.35)
		Over 40			1.03	(0.79–1.33)			0.92	(0.55–1.55)
Total physical activity		First tertile (lowest)			Reference				Reference	
		Second tertile			0.87	(0.75–1.01)^†^			1.08	(0.71–1.64)
		Third tertile (highest)			0.83	(0.71–0.97)^*^			0.99	(0.64–1.53)

CI, confidence interval; PR, prevalence ratio.

^†^; *P* < 0.1, ^*^; *P* < 0.05, ^**^; *P* < 0.01.

In addition, to eliminate the influence of the working conditions, we conducted an analysis of the entire sample stratified by employment status, as displayed in Table 3. Although the results of employment versus non-employment showed almost the same tendency, higher frequency of second-hand smoking was associated with a higher prevalence of depressive symptoms, especially among non-smokers. On the other hand, the point estimate of “almost every day” in non-smokers who were not employed was higher than that in non-smokers who were employed. Among non-smokers who were employed, PRs compared with “almost never” were 1.23 (95% CI, 1.05–1.45) for “sometimes” and 1.31 (95% CI, 0.98–1.75) for “almost every day”; among smokers who were employed, PRs were 0.84 (95% CI, 0.52–1.36) for “almost never”, 1.13 (95% CI, 0.78–1.48) for “sometimes”, and 1.26 (95% CI, 0.98–1.75) for “almost every day”. Among non-smokers who were not employed, PRs compared with “almost never” were 1.30 (95% CI, 1.03–1.64) for “sometimes” and 1.82 (95% CI, 1.01–3.28) for “almost every day”; among smokers who were not employed, PRs were 0.92 (95% CI, 0.49–1.73) for “almost never”, 0.97 (95% CI, 0.49–1.93) for “sometimes”, and 1.21 (95% CI, 0.54–2.73) for “almost every day”.

**Table 3.** PRs and 95% CIs for depressive symptoms, determined by multivariable Poisson regression analysis stratified by employment status

			Employment		Not employment	
	
			PR (95% CI)		PR (95% CI)	
Non-smoker	Second-hand smoking	Almost never	Reference		Reference	
		Sometimes	1.23	(1.05–1.45)^**^	1.30	(1.03–1.64)^*^
		Almost every day	1.31	(0.98–1.75)^†^	1.82	(1.01–3.28)^*^
Smoker		Almost never	0.84	(0.52–1.36)	0.92	(0.49–1.73)
		Sometimes	1.13	(0.82–1.56)	0.97	(0.49–1.93)
		Almost every day	1.26	(0.90–1.75)	1.21	(0.54–2.73)

CI, confidence interval; PR, prevalence ratio.

^†^; *P* < 0.1, ^*^; *P* < 0.05, ^**^; *P* < 0.01.

Adjusted for age, gender, body mass index, educational attainment, marital status, present illness, alcohol consumption, smoking volume, and total physical activity.

## DISCUSSION

In this cross-sectional study, we examined the association between the frequency of second-hand smoking and depressive symptoms among adults from the general Japanese population. The present study revealed a positive association between the frequency of second-hand smoking and the risk of depressive symptoms. This association was significant among non-smokers but not significant among smokers after adjustment for all covariates. The results suggest that second-hand smoking is a risk factor for depressive symptoms. To the best of our knowledge, this is the first study on the association between second-hand smoking and depressive symptoms among Japanese general adults. Our findings highlighted the importance of tobacco control measures in terms of protecting mental health.

Previous epidemiological studies have reported an association between second-hand smoking and poor mental health outcomes, including depressive symptoms among adults,^9^^–^^13^ and we demonstrated similar results among adults from the general Japanese population that support those of earlier studies. However, some observational studies that measured the biomarkers of exposure to second-hand smoke in non-smokers have found no association with mental health, and reported conflicting findings.^18^^,^^19^ We believe that, although biomarker (plasma^18^ or salivary^19^ cotinine levels) assessment of second-hand smoking is valid, it may not necessarily reflect long-term exposure to second-hand smoke. Intermittent exposure to second-hand smoke may alter measured cotinine levels, especially if the frequency of second-hand smoking for several days prior to sampling is low. This might diminish the association between second-hand smoking and mental health, as mental health deterioration probably occurs only after long-term exposure. Because we assessed the frequency of second-hand smoking over the last year, we would have been able to detect an association with mental health, although misclassification may have occurred.

In the present study, we conducted stratified analysis according to employment status because second-hand smoking might be a proxy for working conditions.^29^^,^^30^ In support of this notion, exposure to second-hand smoke was similarly associated with depressive symptoms in both employed and non-employed participants. Therefore, the association was thought not to be affected by the potential impacts of work-related variables, such as work environment or class. However, the point estimates of the risk of depressive symptoms were observed to be somewhat higher among non-employed than among employed individuals. Jung et al reported that, although second-hand smokers both at home and at work had an increased risk of depression, the effect size was greater for second-hand smoking at home than at work.^9^ Second-hand smoking at places other than the workplace, such as at home, might have a greater effect on mental health. However, in the present study, because we have no information about the place of exposure to second-hand smoke, the impacts of second-hand smoking depending on place could not be distinguished. These considerations remain in the realm of speculation.

There are several possible pathways of the association between second-hand smoking and depressive symptoms. First, sidestream smoke might influence the unintended intake of harmful substances. In particular, the intake of nicotine during second-hand smoking could affect dopamine levels in the human brain.^31^ As nicotine addiction dysregulates the dopamine system, which, in turn, increases vulnerability to depression among smokers,^32^ similar effects might occur among individuals exposed to second-hand smoke. Second, inhaling and smelling others’ cigarette smoke might be psychologically stressful. Kim et al^17^ reported that exposure to second-hand smoke was associated with high psychological stress, especially among non-smokers. Therefore, exposure to second-hand smoke might lead to depressive symptoms through increased psychological stress. Third, sleep disturbance caused by second-hand smoking might affect mental health. Several studies have reported an association between second-hand smoking and sleep disturbance,^33^^,^^34^ which might lead to depressive tendencies. However, further research studies are needed to examine the pathway by which second-hand smoking leads to depressive symptoms.

Our results showed that the association between second-hand smoking and depressive symptoms was significant for non-smokers but not for smokers. Non-smokers may be relatively more affected psychologically by second-hand smoking than smokers. A previous study showed an association between second-hand smoke and subjective stress in both smokers and non-smokers, but the effect was greater for non-smokers.^17^ In non-smokers, psychological stress from second-hand smoke may lead to depressive symptoms. In our study, the PR point estimates of depressive symptoms for non-smokers were statistically significant, but those for smokers were not. However, because the point estimates for smokers were comparable to those for non-smokers, we may not have been able to detect the effects of second-hand smoking among smokers owing to the small sample size. Additionally, the cross-sectional design of our study meant that the results concerning the effects of second-hand smoking on smokers and non-smokers and the differences between them are not conclusive. Therefore, further studies using longitudinal study designs with larger samples are needed in the future.

This study has several limitations. First, the assessment of frequency of exposure to second-hand smoke was based on a self-administered questionnaire. Thus, misclassification of the frequency of second-hand smoking might have occurred. However, a previous study demonstrated that self-reported exposure to second-hand smoke could be useful in epidemiological studies,^35^ and the self-report questionnaire is the most commonly used tool for assessing exposure level to second-hand smoke.^36^ Therefore, the effect of misclassification may have been small. Second, we did not have information regarding the places of exposure to second-hand smoke, such as at home or at the workplace. Exposure to second-hand smoke might be a proxy for stressful living and working conditions,^29^^,^^30^ which are potentially related to depressive symptoms. Therefore, in order to eliminate the impact of the working conditions, we conducted stratification analysis according to employment status, which showed nearly consistent results. However, we did not examine the effects of the home environment; therefore, further studies are needed to examine the different effects of the place of exposure to second-hand smoke. Third, our consideration of socioeconomic factors was limited to education and employment. We did not adjust for respondents’ income, because we did not have any data on income. Socioeconomic status has been reported to be related to second-hand smoking^37^^,^^38^ and mental health,^39^^,^^40^ which might have confounded the results. However, we believe that the possible confounding effect of income level may have been controlled because we adjusted for educational attainment and employment status. On the other hand, we were unable to examine how job category (blue-collar or white-collar) might have influenced the association between second-hand smoking and depression, as we lacked the relevant data. Fourth, we were unable to consider the duration of exposure to second-hand smoke. Because the life-course of second-hand smoking (including smoking behavior in childhood and adolescence) may affect mental health, further long-term observation studies are needed. Fifth, although our results suggest that second-hand smoking is associated with a higher risk of depressive symptoms, the small number of second-hand smoking categories (owing to the small sample size) makes it difficult to demonstrate a clear dose-response relationship. Sixth, we do not have any data on participants’ history of depression or medication for mental illness; therefore, we were unable to consider the effects of these factors. Thus, misclassification might have occurred in the assessment of depressive symptoms. Finally, because the present study recruited the participants from the general population who visited the public health center for annual general health check-ups, the participants may have been more concerned about their health than other residents in the area, which may limit the generalizability of our findings.

### Conclusion

In conclusion, the present cross-sectional study showed that second-hand smoking was associated with a higher risk of depressive symptoms. This association was significant among a sample of the non-smoking adult Japanese general population. Our findings suggest that the association between exposure to second-hand smoke and poor mental health outcomes is also applicable to Japanese adults, and underscore the importance of tobacco control measures in terms of protecting mental health. We believe that a total ban on smoking in public venues is necessary, in addition to further efforts to raise public awareness of the dangers posed by second-hand smoking to mental health.
